# Psychometric properties of Lithuanian translation of the self-report version of the Liebowitz social anxiety scale in young adult sample

**DOI:** 10.3389/fpsyt.2026.1787195

**Published:** 2026-04-22

**Authors:** Olga Zamalijeva, Karolina Petraškaitė, Giedrius Rupšys, Ieva Jurevičiūtė, Goda Gegieckaitė, Michael R. Liebowitz, Jonas Eimontas

**Affiliations:** 1 Institute of Psychology, Faculty of Philosophy, Vilnius University, Vilnius, Lithuania; 2 The Medical Research Network, LCC, New York, NY, United States

**Keywords:** confirmatory factor analysis, internal consistency, Liebowitz social anxiety scale, psychometric properties, young adults

## Abstract

**Background:**

Social anxiety disorder starts in adolescence or young adulthood and may have damaging effects on psychosocial development of the individual. Any intervention starts from assessment and the Liebowitz social anxiety scale with later developed self-report version is a valuable tool for practitioners for almost four decades. Even though the original version and adaptations have consistently demonstrated good reliability, there remains considerable debate regarding the factor structure. The aim is to test the factor structure and internal consistency of Lithuanian translation of the self-report version of the Liebowitz social anxiety scale (LSAS-SR) in a non-clinical young adult sample.

**Method:**

Data of 452 young adults (mean age 21.3, 69.7% female) who volunteered participate in the study was used. Two factor solutions were tested: a single-factor model, with anxiety/fear and avoidance ratings loading on one factor, and a higher-order factor model, including two second-order scales (anxiety/fear scale and avoidance scale) and four first-order subscales (social interaction anxiety, performance anxiety, social interaction avoidance, performance avoidance). Internal consistency assessed using Cronbach’s alpha.

**Results:**

Lithuanian version has excellent internal consistency for the total score, scales and subscales, with Cronbach’s alfas ranging.85-.96. Confirmatory factor analysis shows that both tested models have acceptable data fit (RMSEA = .062-.067; CFI = .93-.94), however strong associations between (sub)scales, i.e. correlations exceeding.80, suggests that the use of scale and subscale scores may be less informative, especially in cross-sectional research, but could provide nuanced information in individual assessment.

**Conclusion:**

Further research on psychometric properties of Lithuanian versions of LSAS-SR should focus on verifying these results in a representative sample and in a clinical sample as well as testing the convergent and discriminant validity.

## 1 Introduction

Social anxiety disorder (SAD) is characterized by an overwhelming anxiety/fear of social situations, where there is a potential for evaluation by others, such as during social interactions (e.g., having a conversation) or doing something while feeling observed (e.g., performing a task in front of others) (1), resulting in consistent avoidance of relevant social situations. These symptoms can cause significant negative impact across various aspects of life, such as romantic relationships (2), academic performance (3), and general quality of life (4).

Social anxiety is thought to be a problem of adolescents and young adults because of its early onset and higher prominence in these life stages. The mean age of onset of anxiety disorders overall was found to be 21 (5), whereas the median age of onset for SAD in particular ranges from 11 to 26 years across different countries, and a lower age of social anxiety onset is also associated with higher recurrence of SAD (6). Some studies suggest that social anxiety diminishes with age (7), others argue SAD to be the most persistent of anxiety disorders, with remission rates lower than general anxiety disorder, panic disorder, or phobias (8). This shows that the social anxiety that emerged in adolescence often continues its way into adulthood, making young people the most affected. Pre-pandemic worldwide 12-month prevalence of SAD was 2.4%, with slight variations between high- and low-income countries (6). However, it has been reported that for young people COVID-19 pandemic brought a substantial increase in many psychiatric symptoms, including social anxiety (9, 10), once again bringing the spotlight to this issue.

Both, high social anxiety prevalence and early age of SAD onset, imply a demand for psychometrically adequate social anxiety scales that would be well adapted to different cultures as well as suitable for young adults. The Liebowitz Social Anxiety Scale (LSAS) (11) is one of the most globally used scales for such aims. The scale was initially designed as a clinician-administered instrument (11) that measures anxiety/fear and avoidance in social and performance situations. It was later adapted into a self-report version (LSAS-SR) to facilitate easier administration [e.g., (12–14)]. Studies indicate that the self-report LSAS-SR version closely aligns with the original format, demonstrating strong association with the clinician-administered version (12, 15) and subscale intercorrelations (13).

LSAS-SR demonstrates good reliability, which was explored across various cultural adaptations, such as Spanish (16), French (17), Japanese (18), Portuguese (19), Italian (20), and Indonesian (21). Most studies report strong internal consistency, with Cronbach’s alpha exceeding 0.90 for the total scale and 0.70 or higher for its original subscales (12–14, 16, 17, 19, 21, 22). Previous studies also showed good test–retest reliability (12, 19).

There are numerous studies supporting convergent and discriminant validity of LSAS-SR. Significant associations with other self-report social anxiety scales were reported across various cultural adaptations, including, moderate to strong correlation with Social Phobia Anxiety Inventory (SPAI) (12), moderate to strong correlation with Social Phobia Scale (SPS) (12, 13), correlations with relevant Social Anxiety Questionnaire for Adults (SAQ) dimensions (16). Srisayekti et al. (21) found correlation between LSAS and The Brief Fear of Negative Evaluation Scale (BFNE). The discriminant validity of LSAS-SR has been examined through correlations with measures of depression (13, 16, 17) and anxiety (19), generally showing low to moderate associations. Though convergent and discriminant validity of LSAS in many cases was acceptable, in terms of cultural adaptations testing of these types of validity might be limited due to the lack of other properly validated measures suitable for this purpose, therefore the adaptation often starts with assessment of the factor structure of the newly translated instrument.

The structure of LSAS-SR remains debated, with limited consensus among researchers. Liebowitz (11) originally proposed a four-factor model based on clinical criteria for social phobias (performance anxiety, performance avoidance, social anxiety, and social avoidance). Some studies support the original model (17, 21, 23), however cross-cultural adaptations often report distinct factor structures [e.g., (16, 19, 20)]. Safren et al. (24) and Oakman et al. (14), suggested a four-factor structure, but different factors (social interaction, public speaking, observation by others, and eating/drinking in public). Levin and colleagues (25) proposed three factors that combine social situations related to group activities, dyadic interactions, and individual activities carried out in public. Baroni et al. (20) supported a single‐factor solution arguing redundancy of the four-factor model due to strong associations between them. On the contrary, Baker et al. (12) proposed a five-factor model (social interaction anxiety, nonverbal performance anxiety, ingestion anxiety, public performance anxiety, and assertiveness anxiety), which was later supported by Forni dos Santos et al. (19). Caballo et al. (16) introduced yet another five-factor structure somewhat differing from the Baker et al. (12) model. Notably discussed research has evident variation in sample size (175 – 31–243 subjects), sample type (clinical or non-clinical), age (from teenagers to middle aged adults), analysis strategy (exploratory or confirmatory), different number of items or measures (only 19 items, only anxiety/fear measurement), which may account for same variation in conclusions, but even so results remain greatly contradictory. Despite various proposed alternatives, there is sufficient empirical support for the originally proposed scale and subscale composition that was based on theoretical assumptions and clinical presentation of social anxiety (11), thus it is reasonable to start testing the original factor structure, which includes the overall social anxiety measure, interaction and performance anxiety scales with four subscales. Keeping original factor structure in different language adaptations, provided achievement of sufficient data fit, might be considered preferable as it allows to maintain international comparability in research.

Although raising the question of the consistency of the structure in different contexts, the scale exhibits adequate discrimination between clinical and non-clinical samples (20, 22, 26). Large effect sizes, Cohen’s d >.80, of the differences of subscales of LSAS-SR are found between these samples (16). The total score accurately distinguished individuals with SAD from non-clinical participants and differentiated generalized from non-generalized SAD, with high specificity and sensitivity, results are also comparable between self-repot and clinician administered LSAS (27). Oakman and colleagues (14) demonstrated that the scale and its original subscales reliably discriminate social phobia from panic disorder and obsessive-compulsive disorder. Overall, the findings indicate that LSAS-SR is effective in distinguishing participants with social anxiety from non-anxious controls or from those with other anxiety disorders.

Inconsistent results regarding the factor structure of LSAS-SR across different cultural contexts calls for further studies. Moreover, Lithuanian version of LSAS-SR would facilitate the assessment of social anxiety symptoms within the Lithuanian population that are prevalent in primary care, but remain poorly identified (28), likely due to the lack of properly validated assessment tools. Thus, the current study aims to examine the psychometric properties, namely internal consistency and factor structure, of the Lithuanian version of LSAS–SR.

## 2 Materials and methods

### 2.1 Sample

Data collected from 452 young adults was used in this study. Due to its cost-effective nature, convenience sampling was used. The target group was approached through social media – youth and student Facebook groups. Before providing consent, participants were informed about the study purpose and that the data is collected to evaluate the quality of the Lithuanian version of the social anxiety measure. Participants were informed that participation is anonymous, completely voluntary and they can withdraw consent to participate at any time.

The age range was 18–29 years old. Due to convenience sampling, there is an evident disproportion in gender distribution with women comprising almost 70%. The majority of the sample were students from big cities, and almost half of the participants were in a romantic relationship at the time of data collection. Detailed demographic information is presented in **Table 1**.

**Table 1:** Sample characteristics.

Category	Range	Mean	SD
Age	18–29	21.31	2.57
		N (Total = 452)	%
Age groups	18–19	142	31.4
	20–24	249	55.1
	25–29	61	13.5
Gender	Male	129	28.5
	Female	315	69.7
	Diverse	8	1.8
Place of residence	Big city	368	81.4
	Urban area	40	8.8
	Rural area	44	9.7
Relationship status	Married or cohabitating	111	24.6
	In a relationship	106	23.5
	Single	235	52.0
Highest educational level obtained	Lower secondary education	3	0.7
	Upper secondary education	344	76.1
	Tertiary education (college or university)	105	23.2
Ongoing education	Currently studying	329	72.8
	Currently not studying	123	27.2

### 2.2 Materials

Self-report version of the Liebowitz social anxiety scale consists of 24 different social situations. All items are rated on a four-point scale under 2 conditions: the level of fear or anxiety evoked by the social situation (from 0 – none, to 3 – severe) and the frequency of avoidance of said situation (from 0 – never, to 3 – usually) (29). Based on the original proposal LSAS-SR allows calculating a cumulative score for anxiety/fear and avoidance scales as well as 4 subscales: social interaction anxiety, performance anxiety, social interaction avoidance, performance avoidance. A total LSAS-SR score can also be computed by summing up both, anxiety/fear and avoidance, ratings for all 24 items.

The translation of LSAS-SR to Lithuanian was based on a double-translation and reconciliation strategy (30) and closely followed the procedure described in Walde and Völlm (31). Initial translation was conducted independently by two master’s degree psychology students – native Lithuanian speakers and have very good command of English. After this both translations were reviewed by a panel of 3 researchers with Ph.D. in psychology, where translations were compared and discussed in terms of phrasing, grammar, readability and correspondence to the idea each item represents according to the LSAS Manual (29). As the result of the review and adjudication a proposal for final translation was produced. This version was pretested with 3 students not involved in the translation process, who were asked to rate each statement, to reflect on the meaning of each situation and to provide feedback on phrasing and understanding of each item. As a result, 2 minor clarifications were added: 1) item 3 – an example of a public place; 2) item 7 – possibility of strangers being present. This final version was used for assessment of psychometric properties.

### 2.3 Data analysis

Confirmatory factor analysis (CFA) conducted using Mplus v8.2. Since the items are ranked on a 4-point scale, the weighted least square mean and variance adjusted (WLSMV) estimator was used. A sample of 200–500 participants is considered sufficient for CFA with WLSMV estimator (32). Due to stringent nature χ2 was not used as an absolute fit measure, rather the goodness-of-fit was evaluated based of several indices: RMSEA (root mean square error of approximation), CFI (comparative fit index), TLI (Tucker-Lewis index), SRMR (standardized root mean square residual). According to the most widespread practice, reasonably good fit can be assumed where 1) RMSEA values are close to or below.06, 2) SRMR values are close to or below.08, 3) CFI and TLI values are close to.95 or greater (33).

**Figure 1:**
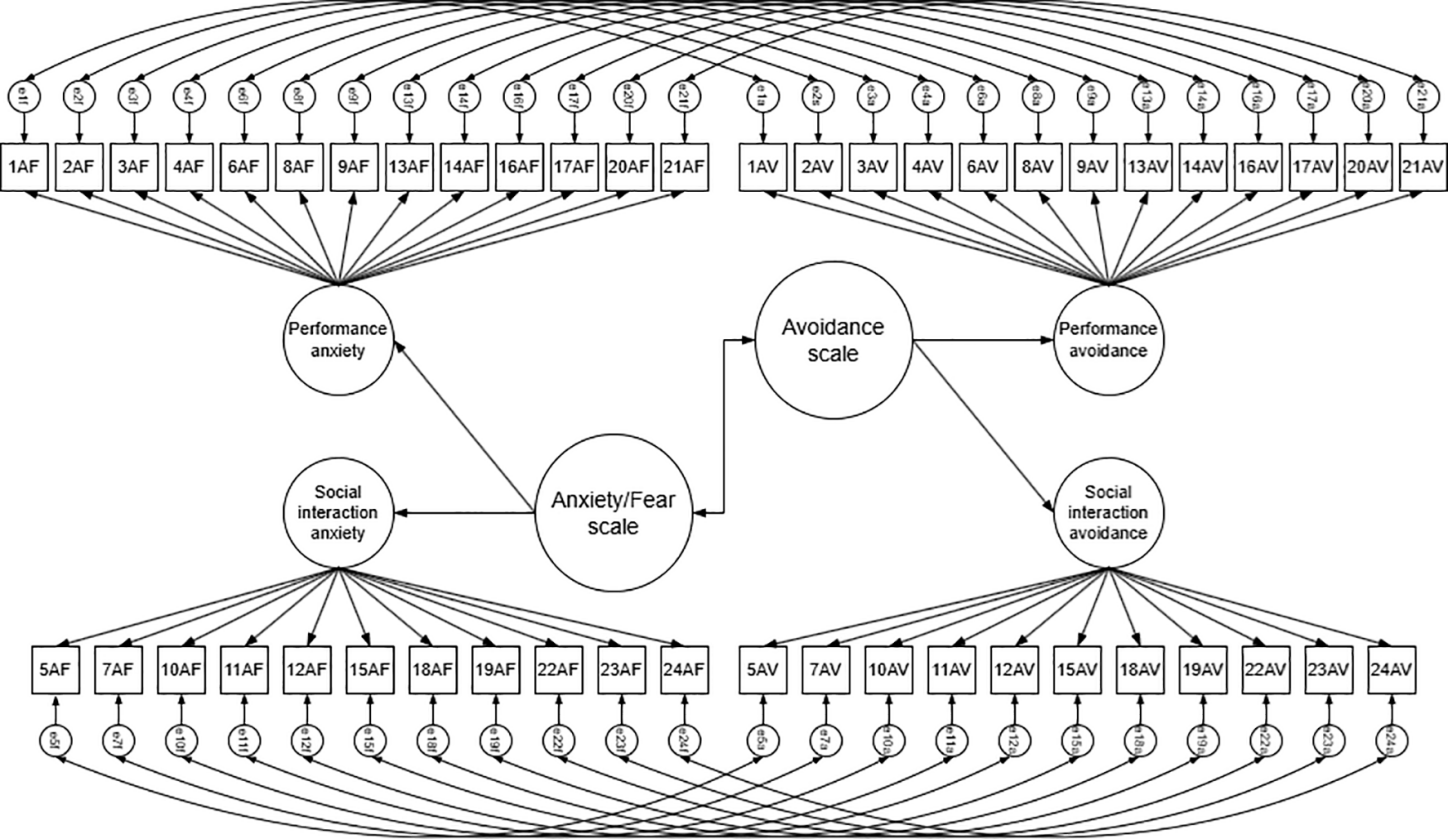
Visual representation of tested higher-order factor model.

In this study 2 factor solutions were tested: 1) single-factor model, where both ratings of all items (48 indicators) are loading on one latent factor; 2) higher-order factor model (**Figure 1**), where social interaction anxiety (11 indicators) and performance anxiety (13 indicators) form LSAS-SR anxiety/fear scale, while social interaction avoidance (11 indicators) and performance avoidance (13 indicators) comprise avoidance scale. Since all the items are rated twice, both tested models include error correlations between two ratings of the same social situation.

Distribution of the data was assessed using skewness and kurtosis. As per common practice the values ±2 are considered acceptable to conclude data distribution close to normal (34).

## 3 Results

Responses showed relatively symmetrical distribution (**Table 2**). The only item that resulted in slight asymmetry in anxiety/fear measurement condition, evident asymmetry in avoidance condition and substantial kurtosis under both measurement condition was item 4. This result may be considered culture specific. Item 4 refers to drinking in public, but does not specify the type of beverages to be consumed. According to Lithuanian legislation, people under the age of 20 are not allowed to consume alcoholic beverages publicly or otherwise and a sizable proportion of subjects (31.4%) in this study are under legal drinking age. Moreover, there are other restrictions concerning alcohol consumption in public that might have also contributed to the deviation from the normal distribution in this particular item.

**Table 2:** Descriptive statistics of the items.

Items	Anxiety/fear			Avoidance			
	*M(SD)*	*Sk*	*Ku*	*M(SD)*	*Sk*	*Ku*
1	1.02(0.92)	0.55	-0.61	1.15(0.93)	0.51	-0.54
2	1.08(0.90)	0.41	-0.68	0.92(0.83)	0.82	0.36
3	0.70(0.91)	1.11	0.23	0.57(0.78)	1.35	1.30
4	0.34(0.66)	1.97	3.38	0.29(0.61)	2.48	6.63
5	1.50(0.92)	-0.03	-0.83	1.04(0.79)	0.57	0.09
6	2.31(0.84)	-1.03	0.22	1.71(1.05)	-0.15	-1.21
7	1.51(1.00)	0.05	-1.05	1.15(0.96)	0.50	-0.66
8	1.69(0.90)	-0.04	-0.88	1.25(0.94)	0.32	-0.79
9	1.13(0.96)	0.50	-0.69	0.84(0.93)	0.96	0.03
10	1.82(0.95)	-0.26	-0.96	1.57(1.02)	0.02	-1.13
11	1.48(0.92)	0.04	-0.83	1.24(0.88)	0.39	-0.49
12	1.57(0.99)	0.00	-1.03	1.19(0.95)	0.42	-0.71
13	0.57(0.83)	1.43	1.35	0.55(0.80)	1.42	1.37
14	1.29(0.98)	0.33	-0.87	0.83(0.93)	0.87	-0.23
15	1.62(1.00)	-0.13	-1.05	1.33(0.98)	0.29	-0.91
16	1.68(0.98)	-0.15	-1.01	1.41(0.98)	0.23	-0.95
17	1.61(0.98)	-0.10	-0.99	0.59(0.82)	1.34	1.15
18	1.45(1.00)	0.03	-1.05	1.29(0.98)	0.33	-0.86
19	1.11(1.04)	0.50	-0.94	1.09(1.02)	0.56	-0.83
20	1.98(0.95)	-0.47	-0.84	1.31(1.07)	0.30	-1.17
21	1.90(1.04)	-0.51	-0.95	1.56(1.11)	0.01	-1.36
22	1.22(1.08)	0.37	-1.15	1.09(1.14)	0.56	-1.15
23	1.45(1.13)	0.05	-1.38	1.39(1.16)	0.17	-1.42
24	1.19(1.00)	0.34	-0.98	1.11(1.08)	0.47	-1.12

Results of CFA show that the data can be considered as consistent with the hypothesized factorial structure of both tested models (**Table 3**). At the same time, none of the models tested resulted in perfect fit (i.e., RMSEA ≤.06, CFI ≥.95, TLI ≥.95, SRMR ≤.08), however all indices are close to the thresholds indicated by Hu and Bentler (33), thus are indicative of acceptable model fit. Furthermore, examination of possible modification indices for single-factor model showed that introduction of error correlations between item 9 and item 8 (under anxiety/fear condition MI = 122.60, under avoidance condition MI = 127.28) as well as item 20 and item 6 (anxiety/fear condition MI = 141.03, avoidance condition MI = 256.14) could result in substantial reduction in χ2 and subsequent fit improvement. In higher-order factor model introduction of error correlations between items 9 and 8 (anxiety/fear condition MI = 102.27, avoidance condition MI = 96.51) as well as item 20 and item 6 (anxiety/fear condition MI = 113.00, avoidance condition MI = 180.80) also might improve model fit. Other modification indices did not show this level of impact on the model fit. The above-mentioned modifications are not only reasonable from statistical perspective, but they also indicate evident relationships between these measures. Items 8 (“working while being observed”) and 9 (“writing while being observed”) both refer to a similar situation of being monitored during a task, while item 6 (“acting, performing or giving a talk in front of an audience”) and 20 (“giving a report to a group”) talk about conduct in front of an audience. If these 4 error correlations were introduced, the data fit would improve in both: single-factor model (χ2(1052) = 2577.97, p <.001, RMSEA = .057, 90% CI [.054,.059], CFI = .952, TLI = .949, SRMR = .062) and higher-order factor model (χ2(1047) = 2366.42, p <.001, RMSEA ≤.053, 90% CI [.050,.056], CFI = .959, TLI = .955, SRMR ≤.059), which would undoubtedly suffice the requirements for good model fit.

**Table 3:** CFA fit statistics for LSAS-SR.

Model	*χ^2^*	*df*	*χ^2^/df*	TLI	CFI	RMSEA	RMSEA 90% CI	SRMR
Single-factor model	3201.33*	1056	3.03	.928	.933	.067	[.064,.070]	.067
Higher-order factor model	2849.56*	1051	2.71	.939	.944	.062	[.059,.064]	.064

* p <.001.

Both tested models showed significant and sufficient factor loadings for almost all indicators (**Table 4**). Despite being significant, the only factor loading lower that.40 was observed in item 13 (avoidance rating). This item is related to public bathroom use (“urinating in a public bathroom”) and is gender-specific, thus lower loading might be related to the current sample characteristics (predominantly female). Similar to other European and Western countries conditions in female bathrooms in Lithuania are more private and are less likely to result in increased avoidance due to social anxiety. At the same time, Baroni and colleagues (20) considered factor loadings greater than.30 to be sufficient since they account for at least 10% of variance.

**Table 4:** Factor loadings for the LSAS-SR.

Item Nr.	Measure	1 factor model	Higher-order factor model			
		LSAS-SR (total)	LSAS-SR social interaction anxiety	LSAS-SR performance anxiety	LSAS-SR social interaction avoidance	LSAS-SR performance avoidance
1	Anxiety/fear	.598		.622		
2		.747		.778		
3		.636		.663		
4		.630		.656		
5		.692	.714			
6		.674		.700		
7		.759	.782			
8		.708		.737		
9		.572		.596		
10		.756	.779			
11		.876	.897			
12		.826	.848			
13		.462		.482		
14		.740		.770		
15		.823	.850			
16		.803		.834		
17		.467		.488		
18		.740	.762			
19		.692	.714			
20		.727		.756		
21		.558		.582		
22		.626	.647			
23		.590	.610			
24		.659	.681			
1	Avoidance	.555				.594
2		.712				.766
3		.626				.673
4		.596				.639
5		.665			.696	
6		.679				.729
7		.661			.692	
8		.630				.677
9		.543				.582
10		.685			.717	
11		.800			.836	
12		.746			.780	
13		.335				.360
14		.646				.695
15		.756			.791	
16		.738				.792
17		.466				.503
18		.611			.639	
19		.613			.641	
20		.702				.752
21		.542				.582
22		.508			.531	
23		.475			.498	
24		.575			.601	
LSAS-SR anxiety/fear scale		–	.975	.964		
LSAS-SR avoidance scale		–			.976	.928

Based on skewness and kurtosis measures the distribution of LSAS-SR scales, subscales and the total score are similar to normal. Internal consistency shows excellent results (alpha ≥.85) on both scale and subscale level. It is also important to note that the correlations between LSAS-SR scales and subscales show very strong associations, where even the lowest coefficients are still exceeding.80. Detailed information on distribution, correlations and reliability is presented in **Table 5**.

**Table 5:** Descriptive statistics, internal consistency and Pearson’s correlations between (sub)scales.

Nr.	(Sub)scale	1	2	3	4	5	6	7
1	Anxiety/fear scale	1						
2	Social interaction anxiety subscale	.967*	1					
3	Performance anxiety subscale	.962*	.860*	1				
4	Avoidance scale	.873*	.844*	.840*	1			
5	Social interaction avoidance subscale	.847*	.869*	.761*	.950*	1		
6	Performance avoidance subscale	.812*	.735*	.834*	.950*	.805*	1	
7	Total LSAS-SR score	.971*	.939*	.933*	.965*	.926*	.907*	1
	Number of items	24	11	13	24	11	13	48
	Cronbach’s alpha	.94	.91	.87	.92	.87	.85	.96
	Mean	33.21	15.92	17.29	26.47	13.49	12.97	59.68
	SD	14.94	8.04	7.46	13.64	7.19	7.16	27.66
	Sk	0.11	0.09	0.20	0.47	0.39	0.57	0.27
	Ku	-0.65	-0.76	-0.50	-0.18	-0.29	-0.06	-0.48

* p <.001.

## 4 Discussion

This study was aiming to evaluate internal consistency and factor structure of the Lithuanian version of LSAS-SR. First, obtained results suggest good internal consistency on scale and subscale level (alpha >.80). Overwhelming majority of reliability analyses also suggest high internal consistency regardless of the sample type (12–14, 16, 17, 19, 21, 22). Of course, some sources mention that alpha greater than.95 (or even.90) is considered excessive (35), while some suggest that.90–.95 is necessary when making important decisions (36). The only scale exceeding the.95 benchmark is the total LSAS-SR score. But since Cronbach’s alpha depends in the number of items (37), there is no surprise that coefficient for the total score, consisting of 48 ratings, is this high. High alpha coefficient may be indicative of redundancy and the overall length of the scale may be less practical in scientific research, however, in terms of clinical practice a broader overview of anxiety inducing situations may be rather useful.

Results of the factor structure analysis show that both tested models show similar fit, that can be considered as acceptable but does not meet all recommended good fit criteria. Unfortunately, due to the nature of the estimator used in the CFA, a simple χ2 difference test is not possible to compare the goodness of fit between two tested models, and the models are too different in structure to apply the DIFFTEST used for comparing CFA results with WLSMV estimator. And since both models fit the data adequately, we cannot confidently reject either factor solution for the Lithuanian LSAS-SR version or even suggest one being better than the other. As mentioned in the introduction, it is difficult to compare results to other research due to variation in methods used, thus results of current analysis will mainly be discussed in the context of studies employing similar analysis strategies (e.g., including all items, allowing correlated errors between anxiety/fear and avoidance measures). Despite having a very modest sample and eventually advocating for the factor solution proposed by Safren et al. (24), results of the analysis performed by Oakman et al. (14) indicate that the four-factor structure based on the original LSAS scoring has the best data fit. Schmits and colleagues (17) performed a comprehensive analysis and concluded that a simple model of four correlated factors fits the data significantly better than other factor solutions, still tested higher-order factor solution showed very similar acceptable fit. Thus, there is support for the four-factor solution not only in the current study, but also in previous research.

On the other hand, it is also important to note that research (14, 16, 17, 21) points out particularly strong correlations between scales and subscales indicating substantial overlap between multiple constructs. High correlation of the anxiety/fear and avoidance factors is observed in the self-report data and investigator administered ratings. Oakman and colleagues (14), who reported high latent correlation between anxiety/fear and avoidance dimensions, suggest that these constructs may not be meaningfully distinct. Similarly, Srisayekti (21) reported very strong correlations in both, two-factor and four-factor models, confirming the idea that anxiety/fear and avoidance are closely linked. Baroni et al. (20) analysis of dimensionality suggests that LSAS-SR could be considered as unidimensional scale. And once again Oakman et al. (14) raises concerns that, although a four-factor model distinguishing between anxiety/fear and avoidance in social and performance situations provides a better fit than other models, the high correlations among dimensions call into question the practical utility of using these factor scores separately. Nonetheless there is value in having an anxiety/fear dimension and an avoidance dimension even if they are often highly correlated. In individual evaluation it serves as a way of both, testing and enhancing the accuracy of responses. It is possible that people undergoing treatment may be pushing themselves to enter more and more feared situations while tolerating continued troubling levels of fear and anxiety, which might affect anxiety/fear and avoidance dimensions differently. And though most SAD intervention studies involve subjects with both, performance and interpersonal anxiety and avoidance, people whose main difficulty is public speaking are yet to be thoroughly studied in terms of treatment. Lastly, results by Schmits and colleagues (2014), who tested the single-factor as well as four-factor models that yielded similar acceptable fit, conclude that (sub)scale scores as well as total LSAS-SR score remain relevant to the scoring system.

### 4.1 Limitations and future directions

Despite the sample size being sufficient for the analysis performed, the sample has some limitations. Convenience sampling resulted in an imbalance of gender proportions, with female subjects comprising almost 70% of the sample. This methodological limitation suggests that the results of this study should be considered with caution as gender differences in social anxiety presentation may influence the factor estimation and the gender imbalance in this sample restricted the possibility to perform the invariance testing, which has been shown to be feasible in anxiety measures in young adult samples (38). Moreover, most study participants were students from big cities, making this sample unrepresentative of Lithuanian young adult population and limiting the possibility of generalization of obtained results. Furthermore, the data was collected in general population, thus it should not be speculated whether the same factor structure would be confirmed in a clinical sample, however, scale properties are similar to the ones obtained in clinical samples [e.g., (14)], which suggests that the scale operates similarly across a range of severities. This study did not assess convergent or discriminant validity, using properly validated tools measuring social anxiety, general anxiety, and depression symptoms, creating another severe limitation to the obtained results. Hence future research should focus on confirming the factor structure of Lithuanian version of LSAS-SR in both, representative and clinical samples, and including external validity measures for a more comprehensive evaluation of psychometric properties. Repeated research suggests that the subscales have very strong intercorrelations, which is rather expected considering the content and scoring procedures of LSAS. On one hand, this might raise ambiguity regarding the practical utility of the subscales, on the other hand, it could be indicative of methodological factors rather than true conceptual redundancy, thus further exploration of such methodological factors could provide additional insight into the performance of the scale. Finally, confirmed internal consistency can be described as excessive (Cronbach’s alpha >.95) suggesting that future studies might consider the possibility of developing a brief LSAS-SR version better suited for research purposes.

## 5 Conclusions

Overall, results suggest that LSAS-SR scales and subscales show very good internal consistency. What is more, both factor models are possible, since single-factor model and higher order-factor model resulted in similar and acceptable data fit. Due to high correlations obtained between the 4 subscales as well as anxiety/fear and avoidance scales, the use of these scores may not be informative in correlational and cross-sectional research, thus the use of total LSAS-SR score may be recommended in this case. However, the scale and subscale scores may provide deeper insight and more nuanced understanding when used in individual assessment or longitudinal research (e.g., some interventions may be more effective at reducing avoidance than anxiety). Summing up, based on the results of this analysis it is recommended to retain original scoring instructions for the Lithuanian version of LSAS-SR.

## Data Availability

The raw data supporting the conclusions of this article will be made available by the authors, without undue reservation.
